# 1-Acetoxypinoresinol, a Lignan from Olives: Insight into Its Characterization, Identification, and Nutraceutical Properties

**DOI:** 10.3390/nu16101474

**Published:** 2024-05-13

**Authors:** Ganesha Yanuar Arief Wijaya, Doretta Cuffaro, Simone Bertini, Maria Digiacomo, Marco Macchia

**Affiliations:** 1 Doctoral School in Life Sciences, University of Siena, 53100 Siena, Italy; g.wijaya@student.unisi.it; 2 Department of Pharmacy, University of Pisa, 56126 Pisa, Italy; doretta.cuffaro@unipi.it (D.C.); simone.bertini@unipi.it (S.B.); marco.macchia@unipi.it (M.M.); 3 Interdepartmental Research Center “Nutraceuticals and Food for Health”, University of Pisa, 56100 Pisa, Italy

**Keywords:** extra virgin olive oil, lignan, 1-acetoxypinoresinol, polyphenols

## Abstract

Extra virgin olive oil (EVOO) is a symbol of the Mediterranean diet, constituting its primary source of fat. The beneficial effect of EVOO is strictly related to the presence of fatty acids and polyphenols, bioactive compounds endowed with nutraceutical properties. Among EVOO polyphenols, lignans possess a steroid-like chemical structure and are part of the phytoestrogen family, which is renowned for its health properties. The natural lignans (+)-pinoresinol and 1-acetoxypinoresinol (1-AP) are commonly present in olives and in EVOO. Although (+)-pinoresinol is found in different edible plants, such as flaxseed, beans, whole-grain cereals, sesame seeds, and certain vegetables and fruit, 1-AP was exclusively identified in olives in 2000. So far, the scientific literature has extensively covered different aspects of (+)-pinoresinol, including its isolation and nutraceutical properties. In contrast, less is known about the olive lignan 1-AP. Therefore, this review aimed to comprehensively evaluate the more important aspects of 1-AP, collecting all the literature from 2016 to the present, exploring its distribution in different cultivars, analytical isolation and purification, and nutraceutical properties.

## 1. Introduction

The Mediterranean diet is renowned for its richness in plant-based foods and is considered healthier than diets rich in animal protein and fat but deficient in fiber that are prevalent in the Western hemisphere. Therefore, countries situated in Mediterranean basins, such as Italy, Spain, and Greece, that used to follow this diet exhibit a lower incidence and lower mortality rates of chronic and degenerative diseases than other Western countries, including the United Kingdom, United States, Norway, and Austria [1].

Several reviews have suggested that the healthful effects of the Mediterranean diet are largely due to the high amount of olive oil used in cooking and meal preparation [2,3,4]. Olive oil has been an important protective food for Mediterranean populations since antiquity (>2000 years) due to its lifelong consumption and numerous beneficial effects on human health, which have been extensively studied for decades [5,6]. In vivo studies have demonstrated that diets rich in olive oil significantly reduce the risk of various cancers [7,8]. Moreover, several papers have revealed a positive association between olive oil consumption and a reduced incidence of chronic and age-related diseases, such as atrial fibrillation [9], dementia [10], type 2 diabetes [11], Alzheimer’s disease [12], and coronary heart disease [13]. The health benefits of olive oil are attributed to the high content of monounsaturated Fatty acids (MUFA) (98–99% of the total weight), e.g., oleic acid and other minor components, primarily polyphenols. Olives and olive-derived products are valuable sources of natural polyphenols, such as secoiridoids (oleocanthal, oleacein), simple phenols (tyrosol and hydroxytyrosol), flavonoids, and lignans. Secoiridoids are the prominent polyphenols present in olive oil, possessing numerous pharmacological properties and conferring the bitterness, pungency, and astringency of olive oil [14,15,16,17,18]. Within its bioactive compounds, olive oil contains lignans, which are the most recently identified phenolic compounds. Brenes et al. [19], using a high-performance liquid chromatography (HPLC) analysis, first proved the presence of lignans in olive oil. The authors identified 1-acetoxypinoresinol (1-AP) and (+)-pinoresinol as the two main lignans contained in olive oil.

Lignans are widely distributed in nature as constituents of many plants. Recently, lignans have been amply studied, becoming an attractive research field. Numerous new lignans are continuously identified in the plant kingdom, with almost two thousand different lignans found in all organisms [20,21,22,23]. Notably, the literature has amply demonstrated that lignans possess considerable health effects, such as anticancer, antioxidant, and antibacterial properties [23]. In particular, 1-AP, unlike other lignans, has not been properly investigated. Whereas abundant works in the literature have described the biological activities and pharmacological properties of (+)-pinoresinol, less information is available on 1-AP. It is exclusively found in olives, in contrast to (+)-pinoresinol, which is identified in many plant species. The concentration of 1-AP is relatively low, ranging from 0.17 mg/kg [24] to 123.5 mg/kg [25]. Thus, the number of studies that discuss 1-AP is limited, and information regarding its distribution, preparative isolation, and biological activities is less detailed in research than (+)-pinoresinol.

This review aims to summarize and comprehensively analyze the distribution, preparative isolation, and biological activities of 1-AP, encompassing the literature from 2016 to the present. Our aim is to provide a comprehensive insight into this undiscovered compound, revealing potential applications for it in contemporary research.

## 2. 1-Acetoxypinoresinol

**Figure 1:**
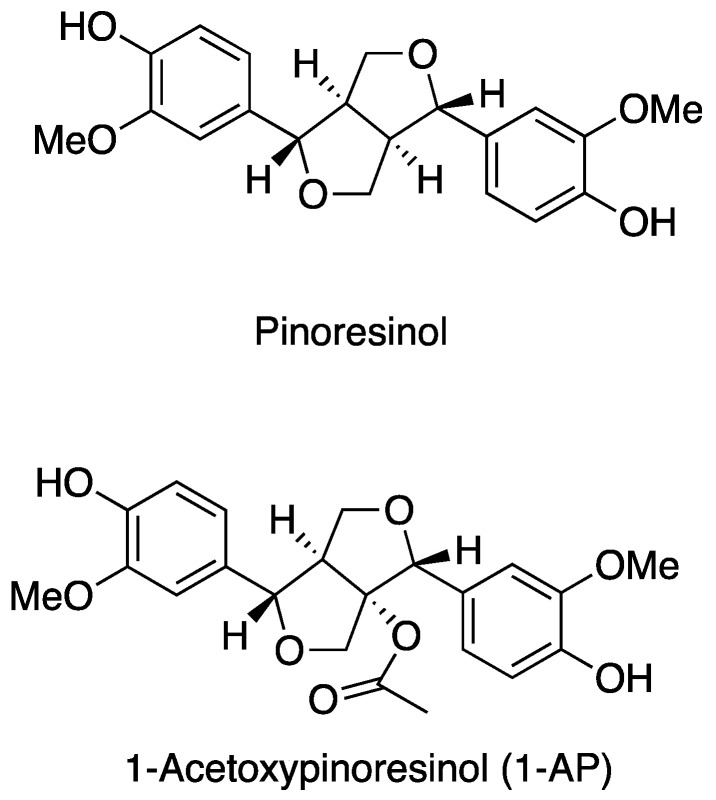
Chemical structure of (+)-pinoresinol and 1-acetoxypinoresinol.

In general, lignans are phenolic compounds with a typical steroid-like structure and are sometimes defined phytoestrogens. The structure of lignans primarily consists of two phenylpropanoid moieties conjugated via their side-chain C8 carbons and occasionally by additional ether, lactone, or carbon bonds. In terms of its chemical structure, 1-AP is very similar to (+)-pinoresinol, differing only by the 1-acetyl substitution (Figure 1). 1-AP is not easily identified, and its characterization requires a combination of different techniques, such as HPLC, NMR, or GC MS analyses. To date, a reliable protocol for the efficient isolation and identification of 1-AP is still lacking due to the absence of a suitable and commercial pure standard. However, considerable efforts have been made within several studies in the past two decades, and the reported techniques for 1-AP analysis are detailed in Section 4, Section 5 and Section 6.

## 3. Distribution of 1-AP on Different Olive Cultivars in Main Producing Countries

As previously mentioned, 1-AP is one of the main lignans present in extra virgin olive oil (EVOO). The concentration of 1-AP, along with other phenolic compounds, in EVOO is strongly influenced by diverse factors (agronomical and technological aspects), such as olive variety, fruit maturation, extraction methods, storage conditions, crop season, geographical area, and environmental conditions [16,26]. Among these aspects, the pressing technology used in olive oil processing is a critical determinant of the polyphenol content, affecting in particular the 1-AP quantity in the final EVOO [27]. As reported by Antonini et al. [28], a two-phase decanter for olive oil production, a recent generation of decanter operated without the addition of water, was able to retain and yield a high concentration of 1-AP in EVOO. In their study, the two-phase decanter extraction effectively increased the concentration of 1-AP in Raggiola olive oil from 19.40 to 39.10 mg/kg and in Leccino olive oil from 14.40 to 18.50 mg/kg, compared to the use of a conventional three-phase separator [28]. Furthermore, in accordance with the study of Ambra et al. [29], the two-phase decanter method successfully preserved higher amounts (1.5 times) of other phenolic compounds (secoridoids, tocopherols, squalene, and carotenoids) and monounsaturated fatty acids than the three-phase decanter. Moreover, the two-phase decanter method is considered more environmentally friendly as it reduces the amount of waste, ensuring more sustainable olive oil production [30]. In fact, the two-phase method produces a new type of olive oil by-product, a sort of wet pomace, called “olive pâté”, containing a high percentage of triacylglycerols rich in oleic fatty acid [31,32].

Among all factors, cultivar and geographical origin significantly influence the different concentrations of 1-AP in EVOO [33,34]. According to Agiomyrgianaki et al. [34], the lignan 1-AP, similarly to the two secorodoids (3,4-DHPA-AC and p-HPEA-EDA) and ligstroside aglycon, is considered a reliable marker to evaluate the quality of EVOO. However, although the concentration of 1-AP is affected by different cultivars, it remains stable during storage [35]. To the best of our knowledge, there are still no reviews focusing on the distribution of 1-AP in different cultivars and geographical locations. An analysis of the existing literature regarding the current distribution of 1-AP in different cultivars of the Mediterranean basin specifying geographical locations is reported in Table 1.

Currently, the top EVOO producers are countries from the Mediterranean area, such as Spain, Italy, and Greece, where different olive species are cultivated. In the global production of olive oil, Spain is the leading producer with a share of 56.2% [45]. In Spain, 1-AP is found at higher levels in several olive varieties, such as Arbequina, Empeltre, and Hojiblanca (Table 1) [19,43], and in particular, Arbequina is reported to be the richest cultivar in 1-AP [19,41,43].

Italy is the second-largest producer of olive oil after Spain, accounting for 24.5% of the world’s total production [45]. In Italy, Puglia and Calabria are the main olive-oil-producing regions, accounting for 68% of the total national production [46]. In Italy, there are about 538 different olive varieties, which offer several flavors and fragrances but also have different compositions. However, data regarding the concentration of 1-AP in Italian cultivars are still limited. Among all the cultivars studied, the Taggiasca cultivar was found to have one of the highest levels of AP (Table 1).

Greece ranks third in olive oil production, accounting for 16.4% of the world’s total production [46]. There are about 20 olive cultivars used for olive oil production in Greece, but the most dominant and important autochthonous cultivars originate from the Peloponnese region. The seaside plains of Lakonia and Messinia in the Southern Peloponnese region offer optimal climate conditions for olive tree cultivation, positively affecting the composition of phenolic compounds in prominent varieties of this area. The distribution of 1-AP in Greek varieties has been studied by Christophoridou’s research group for years. According to Christophoridou and Dais [44], the Koroneiki and Tsunati cultivars contain a high concentration of 1-AP compared to other Greece varieties.

## 4. Extraction of 1-AP from EVOO

1-AP was first identified in olive oil by Brenes et al. [19], and to date, several extraction methods have been developed and validated for a more effective purification process.

**Figure 2:**
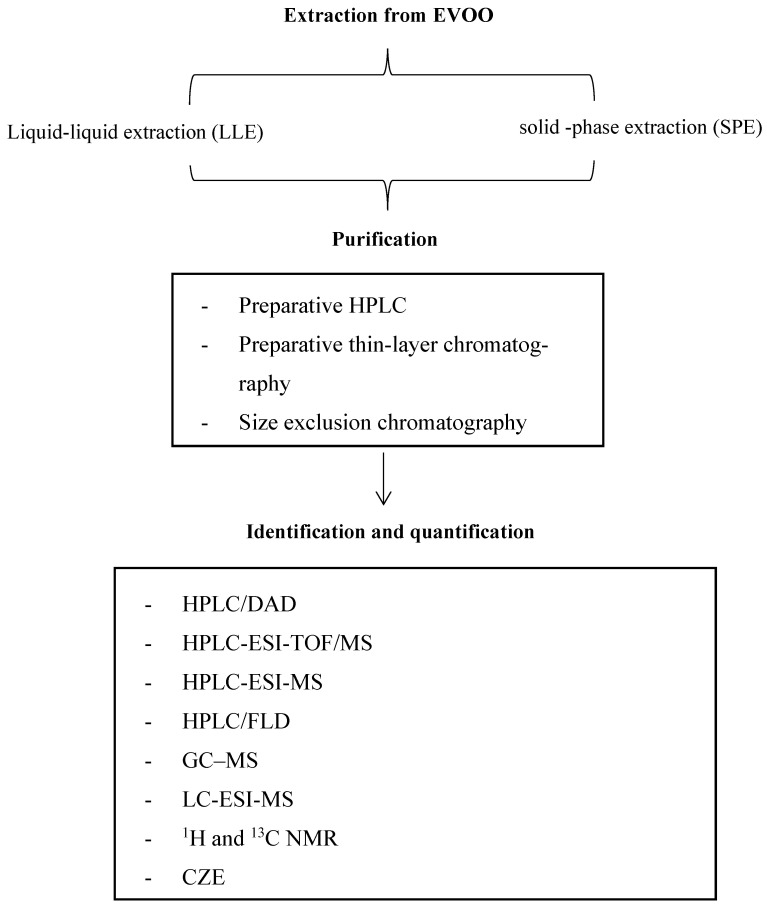
General procedures for the isolation, purification, and characterization of 1-AP from EVOO.

The extraction procedures from EVOO, which are necessary to obtain a sample rich in phenolic compounds for 1-AP isolation, include solid-phase extraction (SPE) and liquid–liquid extraction (LLE) (Figure 2). In several studies, a Diol cartridge for SPE was preferred because of its automated work and shorter extraction time. In general, this solid phase is useful for recovering polar compounds from non-polar matrices. Carrasco-Pancorbo et al. reported SPE with a Diol cartridge (SPE-Diol) for isolating the phenolic fraction from EVOO [47]. Briefly, the EVOO sample was dissolved in hexane and was passed through the Diol column. The cartridge was rinsed with hexane, removing the non-polar fractions. The sample was obtained by washing the SPE with methanol (MeOH) and then evaporating the solvent. SPE-Diol guaranteed extracts from the EVOO in a short period of time (less than 10 min) with an adequate resolution [47]. Nevertheless, in a recent study conducted by Ricciutelli et al. [48], it was demonstrated that the LLE method was more efficient for the extraction of 1-AP than SPE-Diol. The LLE method, employing a mixture of MeOH and N-N dimethylformamide (DMF), was reported to exhibit the highest 1-AP concentration across seven Greek cultivars with an average recovery percentage of over 95% [30].

Capriotti et al. [49] reported that the extraction abilities of LLE and SPE-Diol were similar. However, further evaluation using HPLC-DAD and ultra HPLC-ESI QToF confirmed that LLE provides better recoveries for highly polar, non-polar, and other polyphenols. As a result, the LLE method has been recently used in numerous studies and combined with appropriate detection methods for accurate characterizations of 1-AP. Considering large-scale applications, LLE is also more applicable for industrial uses than SPE.

Moreover, SPE is not preferred because traces of solvent can be sometimes found in the extract as well as the high cost of the equipment [50]. Therefore, LLE is generally reliable for the extraction of 1-AP and is preferred over SPE-Diol due to its simplicity, effectiveness, and lower cost.

The most common LLE technique reported in some studies for the extraction of 1-AP is based on the method by Montedero et al. [51] that uses a MeOH/water mixture (80:20 *v*/*v*). Successively, MeOH has been replaced with acetonitrile (CH_3_CN) because it was demonstrated that MeOH can cause issues in the extraction of EVOO polyphenols. For instance, it can produce the acetalization of secoiridoids, usually extracted with lignans, producing a significant reduction in yield [52]. Therefore, a more recent study reported the isolation and purification of pure 1-AP, starting from commercial EVOO and using the LLE method with CH_3_CN, followed by size exclusion chromatography on Sephadex LH20 [53]. In this LLE procedure, the EVOO was mixed with CH_3_CN, and then the organic acetonitrile layer was subjected to ultra-freezing at 80 °C. The mixture was then immediately filtered to afford a clear oily solution that was then dried and subjected to purification [53].

Another LLE method used water vigorously shaken with EVOO, then immediately filtered and freeze-dried until complete dryness. The filtered aqueous extract was then passed through a resin column, eluted at first with water and then with acetone, and then collected and dried under a reduced pressure [53].

Owen et al. [54] finalized the identification of lignans ((+)-pinoresinol and 1-AP) in EVOO by LLE. First, the olive oil was extracted with MeOH, then the solvent was evaporated and the residue was mixed with CH_3_CN and hexane. The hexane extracts were thrown away, while the acetonitrile solution was evaporated to obtain the extract.

Recently, Rodrigues et al. [55] reported a modern and sustainable method for extracting polyphenols from EVOO using natural deep eutectic solvents (NADES) and evaluated the recovery of various polyphenols, including 1-AP. NADES are green solvents composed of a mixture of two or more components that can form a eutectic mixture with a melting point lower than that of either individual component. These solvents have gained attention due to their low toxicity, biodegradability, and ability to solubilize a wide range of compounds, including polyphenols. In EVOO extraction, NADES serve as an alternative to traditional organic solvents, offering several advantages such as a higher extraction efficiency, reduced solvent toxicity, and environmental friendliness. In this study, NADES, particularly the Xyl/ChCl formulation, proved to be efficient in extracting 1-AP and other phenolic compounds from virgin olive oil. The optimal conditions for extraction were found to be at 40 °C for 1 h with a 1:1 EVOO:Xyl/ChCl-NADES ratio. The solvent can be effectively removed and the phenolic compounds can be concentrated using XAD-16 resin and 100% ethanol as the desorption solution, achieving a 78.3% recovery of 1-AP.

## 5. Purification of 1-AP

For an accurate purification of 1-AP, various techniques such as preparative HPLC [19,35,44], capillary electrophoresis [24], preparative thin-layer chromatography [44,46], and size exclusion chromatography [56] have been adopted.

The first-ever instance of the detection and purification of 1-AP in olive oil was successfully conducted by Brenes et al. [19], through preparative HPLC purification. They used a Spherisorb ODS-2 column and a mobile phase constituted by water and MeOH (90–10), applying a gradient with the MeOH percentage increasing up to 100% in 15 min. In this study, a phenolic extract, obtained from Arbequina olive oils, was chosen. The pure compound, separated through preparative HPLC, was characterized by the combined application of nuclear magnetic resonance (NMR) spectroscopy and mass spectrometry (MS).

Another purification procedure was proposed by Owen et al. [54] and consists of preparative thin-layer chromatography (TLC) using ethyl acetate:isooctane:acetic acid (45:45:10) as a mobile phase. The appropriate bands on the TLC plates detected under ultraviolet (UV) light (254 nm) were scraped and extracted with ethyl acetate. Then, the solvent was evaporated, and the residue was subjected to semipreparative HPLC, which resulted in pure 1-AP with a low yield.

Moreover, Qusa et al. obtained pure 1-AP, starting from EVOO extraction though LLE followed by size exclusion chromatography on Sephadex LH20, using isocratic CH_2_Cl_2_ as a mobile phase [56].

## 6. Identification and Quantification of 1-AP

As reported in the literature, qualitative and quantitative analyses of EVOO phenolic compounds including 1-AP could be carried out using HPLC (coupled with UV, fluorescence, and electrochemical sensors, as well as biosensors and MS detectors) and NMR. Only a few studies have reported the use of gas chromatography (GC) or capillary electrophoresis (CE) [43].

The International Olive Council (IOC), concerning the analysis of EVOO phenolic compounds, agreed with the detection of an hydroalcoholic extract by an HPLC analysis coupled with a diode array detector (DAD). The method adopted a mixture of water (acidified with phosphoric acid), MeOH, and CH_3_CN as a mobile phase and a C18 Spherisorb ODS-2 column as a stationary phase, with a total analysis time of 82 min. Several procedures have been reported in the literature for EVOO phenolic compounds; among them, HPLC coupled with DAD is the most commonly used one for the identification and quantification of 1-AP. Usually, a C18 column, a gradient of water/acetic acid as a mobile phase, and a MeOH mixture are used [57,58]. However, Ricciutelli et al. [48] proved that using a Synergy Polar column for the first time for an EVOO polyphenol analysis offered good resolution and selectivity, representing an effective choice for identifying polyphenol compounds as compared to the conventional C18 columns.

Improvements have been suggested by Bakhouche et al. [59], who performed an analysis of EVOO phenolic compounds, including 1-AP, using a rapid and effective HPLC analysis coupled with an electrospray ionization time-of-flight mass spectrometry (HPLC-ESI-TOF/MS, Bruker Daltonics, Bremen, Germany) system. In this case, the HPLC system was coupled to a time-of-flight mass spectrometer (TOF/MS), equipped with an electrospray ionization source (ESI). In another study, the analysis of 1-AP and other EVOO polyphenols was performed by HPLC coupled with electrospray ionization mass spectrometry (HPLC-ESI-MS) in the negative-ion mode [30]. 1-AP was also identified by HPLC using a fluorescence detector (HPLC/FLD) analysis [28].

Besides HPLC, GC in combination with MS is also used for 1-AP identification, as reported in several studies [25]. García-Villalba et al. investigated the use of GC–MS with a recently developed atmospheric-pressure chemical ionization (APCI) source [35]. Even though the results derived from GC analyses are quite interesting, they are less commonly used due to the required derivatization and the high temperature, which could be harmful for the analytes.

Moreover, an interesting detection method using ^1^H NMR spectroscopy has been proposed by Christophoridou [44]. This technique does not require the prior separation of components; is rapid, quantitative, and non-destructive; and has been proven to be effective in detection of 1-AP in EVOO. In fact, recently, ^1^H NMR spectroscopy has been employed in the analysis of complex mixtures and does not necessitate the separation of individual components [60].

Moreover, ^1^H NMR spectroscopy has been developed for the detection of polyphenols in olive oil and applied to olive drupes [61,62]. Within this method, several compounds can be detected in a single measurement, without the need to pre-treat samples. Furthermore, the ^1^H NMR spectroscopy method does not require internal standards, which might not be commercially available, or derivatization. The detection of EVOO phenolic constituents was based on chemical shifts. For example, 1-AP was identified though a signal at 6.89 δ, six multiplets in the aromatic region, and seven signals in the aliphatic region [63]. Moreover, the ^1^H–^13^C correlation data allowed the authors to unequivocally describe all protons and protonated carbons. It is important to highlight that NMR combined with solid-phase extraction and automation will be permitted when conducting online screenings of a large number of samples in a short period of time.

In a more recent study by Olmo-García et al. [64], a direct injection of EVOO, dissolved in acetone, without any prior extraction or separation process, was performed for the first time on six varieties of Moroccan olive oil by using LC coupled with ESI-MS. The results were reliable, and the quantification of 1-AP was successfully demonstrated and validated by this technique.

Other studies describe the determination of 1-AP and other phenolic compounds present in EVOO through the capillary zone electrophoresis (CZE) method [24,47,56,65]. Specifically, the paper by Caravaca et al. [24] reports a qualitative and quantitative CZE analysis of 1-AP and other phenolic compounds present in EVOO extracts obtained from seven different EVOO Spanish varieties using Diol-SPE.

Moreover, Carrasco-Pancorbo et al. [47,65] proposed for the first time an analytical method relating SPE and CZE coupled with MS for the identification and characterization of phenolic compounds in EVOO. As a detection method, separation by CE coupled with MS exploits the high separation abilities of CE and the potency of MS for the identification of analytes. Meanwhile, ESI is an extremely helpful method coupling with electrophoretic separation. Therefore, the authors developed the first simple SPE–CE–ESI–MS method [47] and then a CZE method coupled with ESI–TOF–MS [65] for the characterization of phenolic compounds in EVOO.

## 7. Nutraceutical Properties of 1-AP

The beneficial effects of olive oil lignans are deeply studied and investigated. Although the health effects of (+)-pinoresinol have been reported by many studies in the literature [66,67,68], only a few studies have investigated the beneficial properties of 1-AP. In fact, experimental data on 1-AP are reasonably limited, as it is not commercially available, and its isolation and synthesis are difficult. Table 2 summarizes the beneficial effects of 1-AP reported in the literature to date based on a few recent studies.

### 7.1. Antioxidant Effect

There is scientific evidence that 1-AP possesses potential antioxidant activity. A study carried out by Morelló et al. [69] reported that 1-AP exhibits a high antioxidant activity toward the oxidation of liposomes (a polar lipid model) and bulk lipids (a nonpolar lipid model). Furthermore, 1-AP demonstrated a slight increase in oxidative stability at a higher concentration of samples (200 mg/kg oil) [74]. Owen et al. [71] tested individual components isolated and purified from EVOO by an in vitro antioxidant assay and compared these to a reference antioxidant, Trolox, and DMSO. 1-AP produced a potent response against the development of reactive oxygen species (ROS) in salicylic acid in hypoxanthine/xanthine oxidase assays, with an IC_50_ value of 0.91 [71]. Moreover, the authors demonstrated the dual action of lignans ((+)-pinoresinol and 1-AP) as donators of the HO- radical and inhibitors in a xanthine oxidase assay [71]. Through a 2,2-Diphenyl-1-picrylhydrazyl (DPPH) assay, both lignans ((+)-pinoresinol and 1-AP) resulted in antioxidants, although the activity of (+)-pinoresinol is higher than that of 1-AP [47]. This behavior could be explained by the presence in 1-AP (though not in (+)-pinoresinol) of the –COOCH_3_ group, which, not being an electron donor, may be responsible for the reduced antioxidant capacity of 1-AP in the DPPH assay [47]. However, these authors demonstrated that both lignans, but in particular, (+)-pinoresinol, had a pro-oxidant activity when used in a lipid model system (OSI), probably due to the oxygen atoms in each central ring of both molecules, which could generate the opening of the ring under the thermal conditions used in the assay. Nevertheless, the –COOCH_3_ group of 1-AP could delay the opening of the ring, resulting in a weaker effect [47].

### 7.2. Antidiabetic Effect

In recent years, the valorization of food by-products as a source of crucial polyphenolic bioactive compounds has been reported [75]. In a more recent study by Mwakalukwa et al. [73], it has been demonstrated that isolated 1-AP from olive mill waste-water (OMWW) has played an important role in reducing the risk of diabetes mellitus. Postprandial hyperglycemia (PPHG) contributes to the development of macrovascular complications, such as cerebrovascular and cardiovascular diseases [76]. By controlling PPHG, diabetes and its complications might be prevented. Carbohydrates are subjected to hydrolysis catalyzed by the enzymes α-glucosidase and α-amylase, leading to an increase in blood glucose levels postprandially. Therefore, the inhibition of the pancreatic α-glucosidase and α-amylase results in a substantial reduction in PPHG blood levels. 1-AP showed high activity against α-glucosidase (IC_50_ = 313.1 vs. 323.4 μM acarbose, the standard drug) and α-amylase (IC_50_ = 13.9 vs. 15.0 μM acarbose, the standard drug). Specifically, it acts as a partial uncompetitive inhibitor to the α-glucosidase enzyme [72]. The inhibitory activity of (+)-pinoresinol was very weak (IC_50_ > 500 μg/mL vs. both enzymes), probably because the presence of –COOCH_3_ in 1-AP increases its α-glucosidase and α-amylase enzymatic inhibitory activities [73]. These results demonstrated the potentiality of this compound in the prevention and/or treatment of type 2 diabetes.

### 7.3. Other Activities

Recently, a study by Qusa et al. [56] demonstrated that 1-AP can reverse and minimalize the fatality of neurotoxicity caused by Penitrem A (PA), a diterpene alkaloid produced by several fungal species. In fact, it is well known that PA can act as an antagonist in brain cells, causing motor system dysfunctions. Both in vitro and in vivo experiments have revealed that 1-AP mediates the PA neurotoxicity mechanism by distorting the Janus kinase (JAK) pathway in Schwan cells and enhancing the recovery of behavioral functions after low-dose PA exposure in a Swiss albino mouse model [56]. These findings can represent a promising solution to prevent food contamination caused by mycotoxins, which often happens in humans and grazing animals because their toxicity can cause the partial or total loss of motor functions by attacking the nervous system. However, it is still too early to apply this finding in medical treatment because 1-AP only acts as a protective agent rather than a regenerative agent against PA toxicity. Therefore, further studies are necessary to combine 1-AP with other medical treatments.

According to Mwakalukwa et al. [72,73], 1-AP could not only be recovered from EVOO but also from its by-products. In OMWW extract, a high level of 1-AP is associated with anti-allergic activity. Their study used the degranulation model on rat basophil leukemia cells (RBL-2H3), demonstrating that the isolated 1-AP from OMWW reduced intracellular Ca^2+^ levels and the expression of calcium channel proteins in RBL-2H3 cells, reducing degranulation. These findings by Mwakalukwa’s group are promising for the development of inexpensive drugs to combat allergies and diabetes using 1-AP derived from OMWW.

Given the literature, plant lignans are known to have beneficial effects on human health, including as anti-inflammatory agents. Previously, it was reported by During et al. [77] that several other plant lignans (secoisolariciresinol diglucoside, secoisolariciresinol, (+)-pinoresinol, lariciresinol, matairesinol, and hydroxymatairesinol) possess anti-inflammatory properties in intestinal cells. Furthermore, it was found that (+)-pinoresinol exhibited anti-inflammatory properties among all the plant lignans tested. However, there are still no studies investigating the anti-inflammatory properties of 1-AP in human intestinal Caco-2 cells.

In another review by Peterson et al. [78], there is much evidence that other plant lignans decrease coronary heart disease and cardiovascular disease. However, there are still no studies investigating the effects of 1-AP intake on the prevalence of cardiovascular disease. Thus, it is important to conduct population studies to study 1-AP’s effects.

Furthermore, metabolism in the human intestine regarding 1-AP remains unknown. Previously, it has been reported that other plant lignans are mostly converted by the intestinal microflora and bacteria to hormone-like compounds called enterolignans (mammalian lignans). The enterolignans then act as a natural cancer-protective compound due to their ability to modulate hormone concentration and influence malignant cell proliferation, differentiation, cell adhesion, and angiogenesis, thus furthermore preventing the development of cancer in the promotional and initiation phase [79].

To the best of our knowledge, there are still no studies focused on the investigation of the metabolism of 1-AP in the human intestine, especially whether 1-AP is converted to enterolignans by human bacteria similarly to other lignans. The result of such a study would be beneficial as key information to utilize 1-AP in pharmacognosy in the future.

## 8. 1-AP as Marker of EVOO Quality

As a lignan exclusively found in EVOO, 1-AP can be used as a marker to authenticate EVOO and to distinguish EVOOs from different cultivars or geographical locations. The authentication of EVOO by using 1-AP has been studied by several authors [43,58,80,81].

According to Brenes et al. [43], 1-AP is a reliable indicator as a varietal marker to discriminate one monovarietal EVOO from another, because the concentration of 1-AP remains stable during storage up to 1 year. In contrast, other phenols may not be suitable for EVOO authentication because of their degradation during storage. In a study by García et al. [58], the aomunt of 1-AP was higher in all samples from the Arbequina cultivar compared to that of those from the Picual cultivar. Thus, 1-AP was used to distinguish olive oil samples from these two cultivars. In terms of geographical location, a high concentration of 1-AP was reported by Nescateli et al. [82] to be a significant indicator to differentiate olive oil from the Sabina PDO (protected denomination of origin) from olive oil from other geographical locations.

Beyond its benefits for human health, there is also evidence that 1-AP possesses benefits for agriculture. In a paper by Kadowaki et al. [79], it was demonstrated that 1-AP stimulated insects’ feeding behavior, especially olive weevil (*Dyscerus perforatus*). It has been observed that 1-AP plays an important role in the preferential stimulation of the female weevil. Thus, this finding could lead to the potential use of olive trees containing a significant amount of 1-AP as lures to attract olive weevils to specific areas.

## 9. Conclusions

The Mediterranean diet is known for its high intake of plant-based foods and EVOO, which has been linked to a range of health benefits, including a reduced risk of chronic and degenerative diseases. The health benefits of EVOO are mainly due to its rich polyphenolic composition, which includes secoiridoids, simple phenols, flavonoids, and lignans. Of these, 1-AP is noteworthy for its exclusive presence in EVOO and its potential health-promoting properties.

This review comprehensively analyses the distribution, preparative isolation, and biological activities of 1-AP, collecting all of the literature from 2016 to the present. The structure and properties of 1-AP are discussed, highlighting its similarity to (+)-pinoresinol and the challenges associated with its identification and characterization due to the lack of commercial pure standards and reliable analytical protocols.

The distribution of 1-AP varies among different olive cultivars and is influenced by various factors, such as olive variety, fruit maturation, extraction methods, storage conditions, and geographical origin. Various extraction and purification methods have been developed and validated for the isolation of 1-AP from EVOO, including SPE and LLE followed by preparative HPLC. Among these methods, LLE has been reported to be more efficient and cost-effective for large-scale applications. The biological activities of 1-AP have been explored in several studies, revealing its potential anticancer, antioxidant, and antibacterial properties. However, further research is needed to fully understand the mechanisms underlying these beneficial effects and to explore its potential therapeutic applications in the prevention and treatment of various diseases.

In conclusion, 1-AP, a unique lignan found exclusively in EVOO, holds promise as a bioactive compound with potential health benefits. Its distribution in different olive cultivars and its extraction and purification methods have been comprehensively reviewed in this paper. Future research should focus on elucidating the biological mechanisms of 1-AP, further exploring its potential therapeutic benefits in promoting human health and preventing chronic and degenerative diseases. Incorporating olive oil, rich in 1-AP, into daily dietary habits, particularly in the context of a Mediterranean diet, could contribute to improving public health and reducing the burden of chronic diseases.

## Figures and Tables

**Table 1:** The distribution of 1-AP in different cultivars of Mediterranean regions.

Cultivar	Geographical Origin	Concentration (mg/kg)	References
Taggiasca	Liguria, Italy	48–160	[36]
Leccino	Marche, Italy	14.40–18.40	[28]
Raggiola	Marche, Italy	19.40–39.10	[28]
Ascolana Tenera	Marche, Italy	5.98–8.94	[37]
Coroncina	Marche, Italy	5.52–8.86	[37]
Mignola	Marche, Italy	8.13–10.30	[37]
Piantone di Mogliano	Marche, Italy	5.31–9.03	[37]
Raggia	Marche, Italy	31.77–35.47	[37]
Canino	Marche, Italy	2.0–2.9	[38]
Coratina	Puglia, Italy	3.6–33.9	[39,40]
Nocellara	Puglia, Italy	2.7–26.2	[39,41]
Ogliarola	Puglia, Italy	2.9–4.3	[39]
Peranzana	Puglia, Italy	3.6–3.9	[39]
Bosana	Sardinia, Italy	7.5–14.8	[40]
Semidana	Sardinia, Italy	10.9–49.4	[40]
Maiorca	Sardinia, Italy	19.9	[40]
Paschixedda	Sardinia, Italy	21.5	[40]
Terza Grande	Sardinia, Italy	23.3	[40]
Terza Piccola	Sardinia, Italy	22.5	[40]
Corsicana da Olio	Sardinia, Italy	56.9	[40]
Pizz’e Carroga	Sardinia, Italy	17.5	[40]
Sivigliana da Olio	Sardinia, Italy	36.5	[40]
Frantoio	Tuscany, Italy	23.1	[40]
Seggianese	Tuscany, Italy	40	[36]
Moraiolo	Tuscany, Italy	8.3–11.5	[38]
Sikitita	Cordoba, Spain	8.27	[42]
Arbosana	Cordoba, Spain	11.70	[42]
Changlot Real	Cordoba, Spain	7.00	[42]
Arbequina	Catalonia, Granada and Cordoba, Spain	26.1–66.9	[19,41,43]
Empeltre	Huezca, Spain	31.5–94.2	[19,43]
Hojiblanca	Lucena, Spain	24.4–50.1	[19,41,43]
Picudo	Jaen, Spain	6.8–12.5	[19]
Cornicabra	Jaen, Spain	10.2	[19]
Picual	Jaen, Granada and Cordoba, Spain	1.9–16.9	[19,41,43]
Athinolia	Lakonia, Greece	2.29–11.10	[44]
Koroneiki	Messinia, Peloponnese, Greece	20.1–26.2	[44]
Tsunati	Messinia, Peloponnese, Greece	21.3–28.7	[44]
Athinolia	Lakonia, Greece	2.29–11.10	[44]

**Table 2:** Recent updates on the beneficial effects of 1-AP.

Nutraceutical Effect	Biological Process	References
Antioxidant	Antioxidant activity toward oxidation of liposomes and bulk lipids	[69]
	increase in oxidative stability	[70]
	potent response against ROS attack	[71]
	DPPH assay	[47]
Protection against neurotoxicity	Mediation of PA toxicity on Schwann cells	[56]
Anti-allergic	Decrease in the expression of calcium channel proteins in RBL-2H3 cells	[72]
Anti-diabetes	Inhibitory activity towards α-glucosidase and α-amylase enzymes	[73]
